# Extranodal marginal zone lymphoma presenting as a skin-based breast finding detected on screening mammography

**DOI:** 10.1016/j.radcr.2026.01.060

**Published:** 2026-02-20

**Authors:** Yu-Soon Aileen Park, David J. Supeck, Alan Zhu, Laura Harper, Richard E. Sharpe

**Affiliations:** a Division of Breast Imaging and Intervention, Mayo Clinic Arizona, 5881 E. Mayo Blvd, Phoenix, AZ 85054, USA; b Mayo Clinic Alix School of Medicine, 13400 E Shea Blvd, Scottsdale, AZ 85259, USA

**Keywords:** Breast lymphoma, Extranodal marginal zone lymphoma, Dermal lesions, Tangential mammography, Breast ultrasound, BI‑RADS

## Abstract

Extranodal marginal zone lymphoma (EMZL) of the breast is rare and may mimic benign skin lesions at screening. We report a 72-year-old woman with a new superficial mass on screening mammography. Tangential views and ultrasound localized the abnormality to the dermis/subdermis, and ultrasound-guided biopsy revealed a low-grade B cell lymphoma with plasmacytic differentiation. Staging with PET/CT, a skin punch biopsy of a cutaneous shoulder lesion, and bone marrow biopsy confirmed disseminated disease, consistent with stage IV EMZL. This case underscores practical imaging steps, strategies, and differential diagnostic considerations to correctly identify skin-based findings and avoid misclassification. When appearances are new or atypical, particularly without a visible cutaneous correlate, tissue sampling is warranted to exclude malignancy, including dermal lymphoma.

## Introduction

Extranodal marginal zone lymphoma (EMZL) of the breast is uncommon [1] and readily masquerades as benign skin disease at screening. Mammographers often downgrade superficial findings when a cutaneous correlate is visible, yet a proportion of “skin-based” densities are not simple nevi or cysts, warranting targeted work-up [2,3]. We report a screening-detected superficial mass without a cutaneous correlate, localized to the dermis/subdermis by tangential mammography and ultrasound, which revealed EMZL on biopsy. This case illustrates: (1) a practical stepwise approach to confirming a dermal origin (BB marker, tangential views, ultrasound), (2) imaging and procedural nuances for safe biopsy of subdermal targets, and (3) current staging considerations including the role of FDG PET/CT in EMZL. By highlighting specific pitfalls and a concise differential for skin-based breast findings, we aim to reduce false reassurance and delayed diagnosis.

## Case presentation

**Fig. 1:**
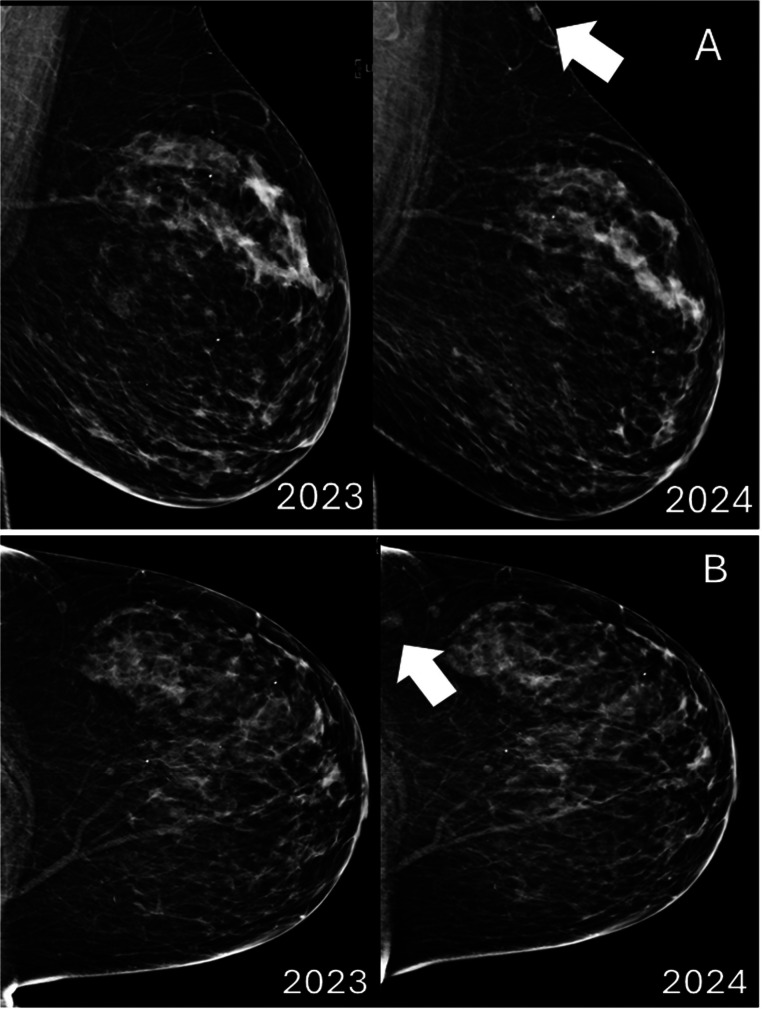
New superficial mass seen in 2024 on routine screening mammogram. (A) Left MLO view demonstrates a superficial mass in the upper posterior breast and (B) Left CC view demonstrates a new mass in the far outer far posterior breast. Both findings are new since the prior study.

An asymptomatic 72-year-old woman underwent her annual screening mammogram demonstrating a new mass in the left breast (Fig. 1). A BI-RADS Assessment Category of 0 (Needs Additional Imaging) was assigned, and the patient was recalled for additional imaging with mole markers placed over any skin lesions after inspection. No obvious cutaneous lesion was visualized over the area of concern; however, there was a small palpable lump near the low axilla, which was identified and marked with a skin BB marker.

**Fig. 2:**
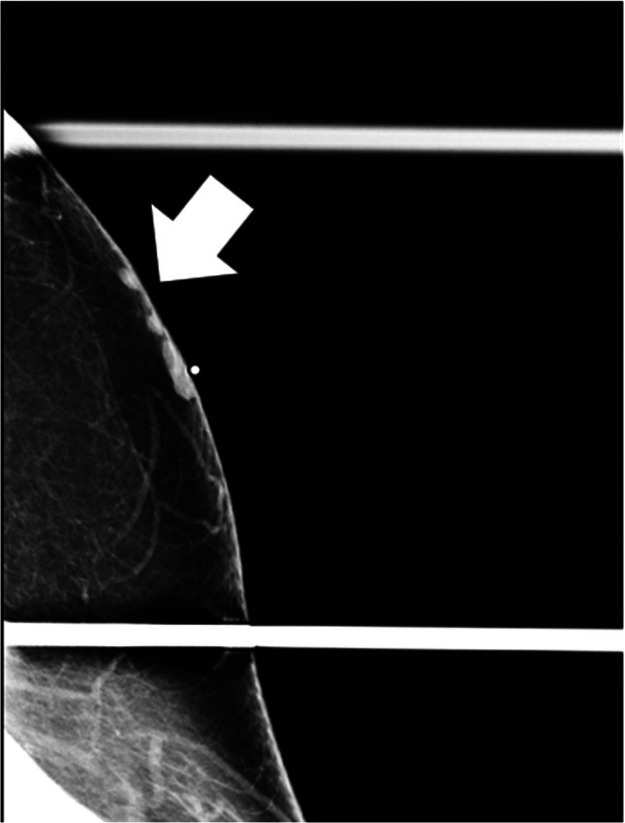
Diagnostic left tangential MLO mammogram obtained after placement of a BB skin marker at the area of palpable lump in the low axilla demonstrates several small superficial masses in continuity with the skin, which correspond to the findings seen on screening mammogram.

**Fig. 3:**
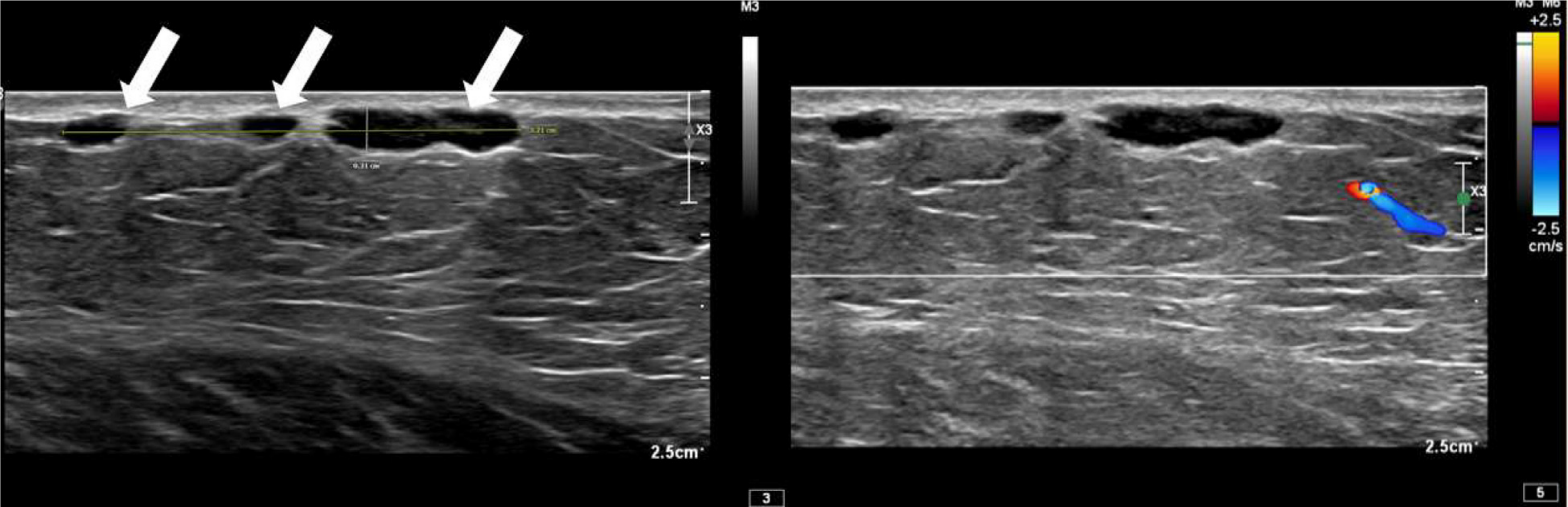
Transverse gray scale ultrasound of the area of palpable concern in the left low axilla demonstrates lobulated, circumscribed subcutaneous masses without internal vascularity, collectively measuring 32 × 3 × 9 mm.

Diagnostic mammogram demonstrated 3 adjacent underlying masses in the skin (Fig. 2). Left breast ultrasound demonstrated 3 corresponding adjacent superficial masses in the left low axilla (Fig. 3). Given the new mammographic finding, absence of a definite benign correlate, and indeterminate sonographic appearance, BI-RADS 4 was assigned, and biopsy was recommended.

**Fig. 4:**
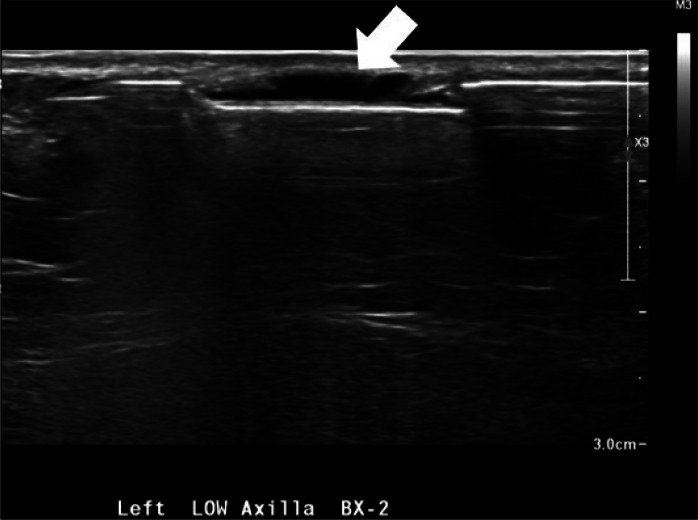
Ultrasound guided biopsy and post procedure mammogram. Ultrasound guided biopsy of one of the 3 hypoechoic lesions in the left subdermal low axillary region using a 12G needle. A ribbon-shaped biopsy clip was placed after sampling (not pictured).

**Fig. 5:**
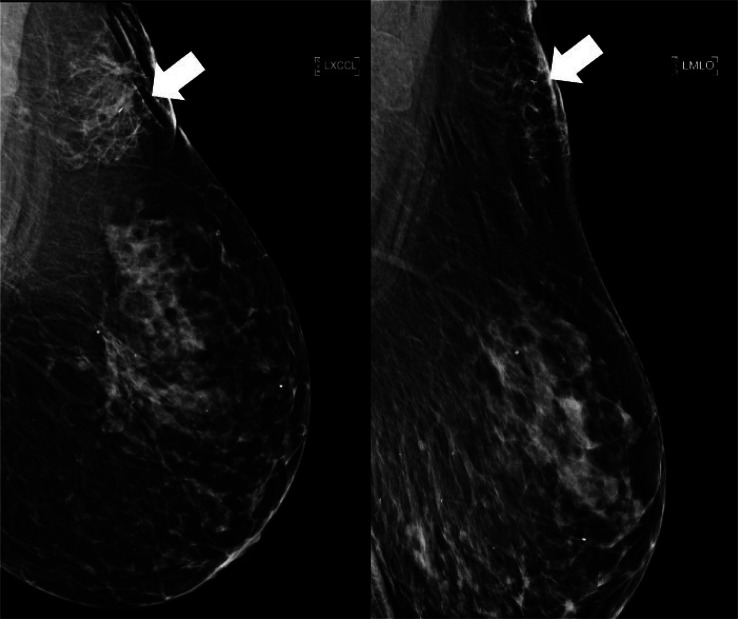
Postprocedure mammogram exaggerated craniocaudal and mediolateral oblique views of the left breast demonstrate expected postprocedure changes and placement of a ribbon-shaped biopsy clip within the targeted finding.

**Fig. 6:**
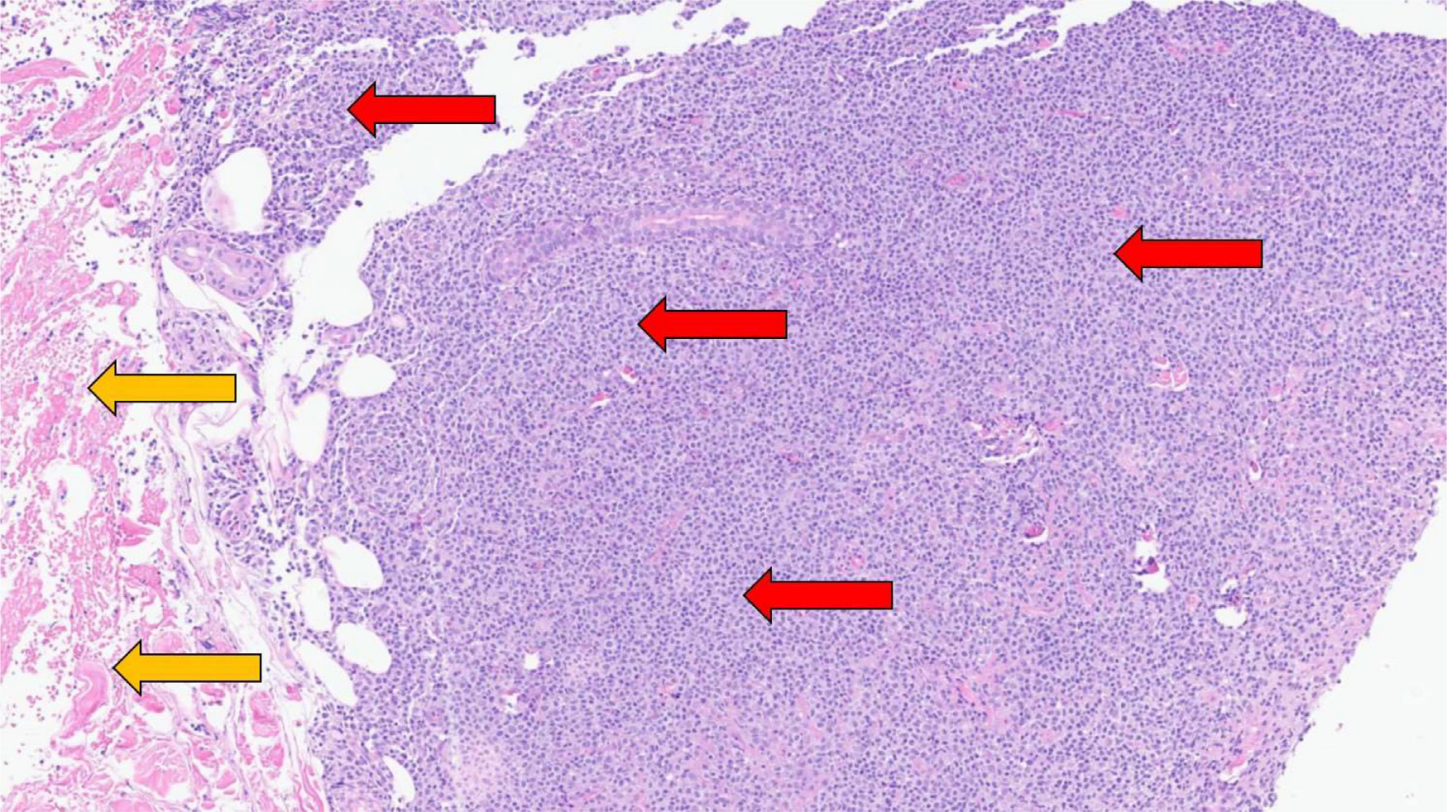
Histopathologic image from the ultrasound-guided biopsy of the left breast demonstrates low-grade B-cell lymphoma (red arrows). Yellow arrows indicate normal dermal tissue.

**Fig. 7:**
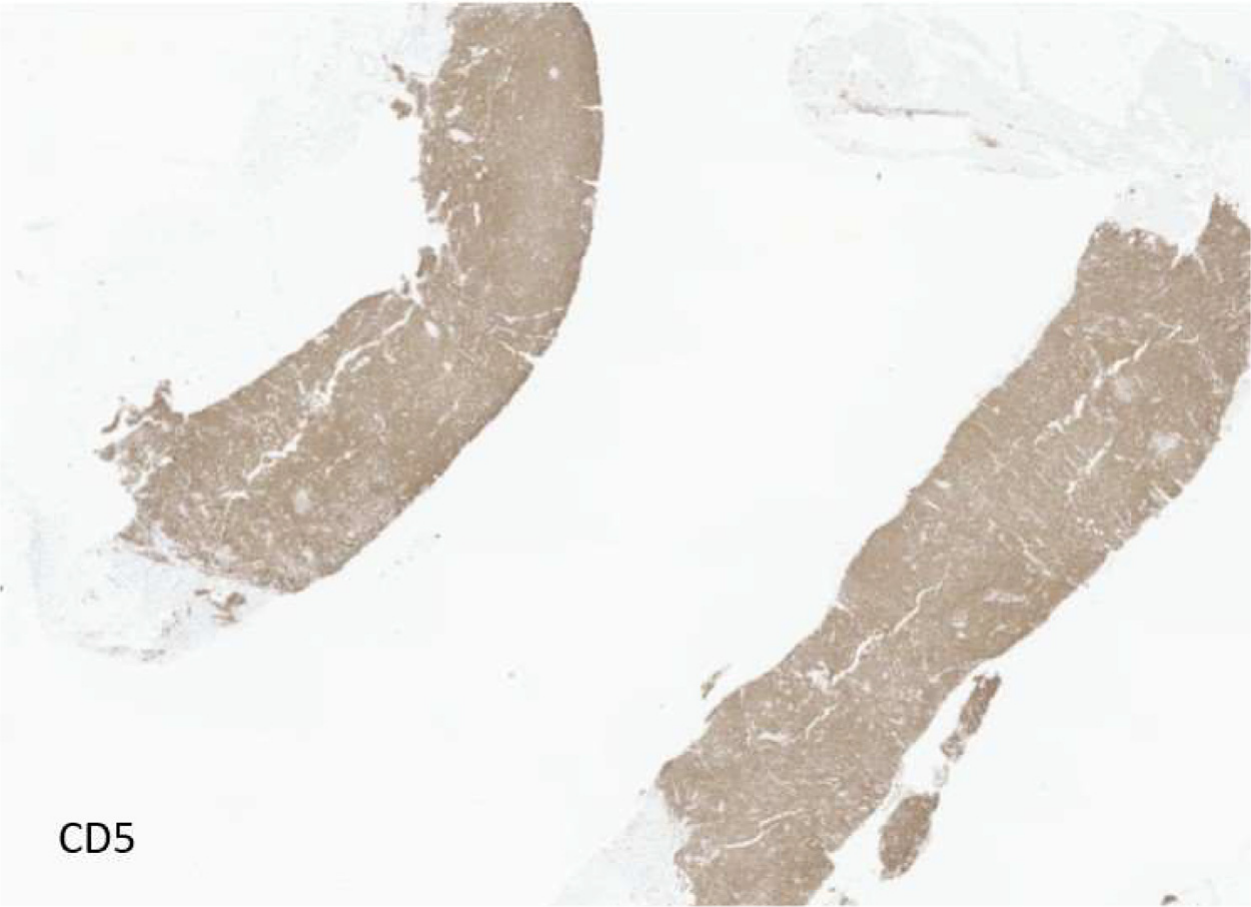
Immunohistochemical staining demonstrates CD5-positive neoplastic B lymphocytes from the ultrasound-guided biopsy of the left breast.

Ultrasound guided core biopsy was performed using an approach parallel to the skin to minimize risk of dermal perforation, a biopsy marker was placed, and postbiopsy mammogram was performed (Fig. 4, Fig. 5). Pathology revealed CD5-positive low grade B cell lymphoma with plasmacytic differentiation, consistent with malignancy and concordant with imaging (Fig. 6, Fig. 7).

**Fig. 8:**
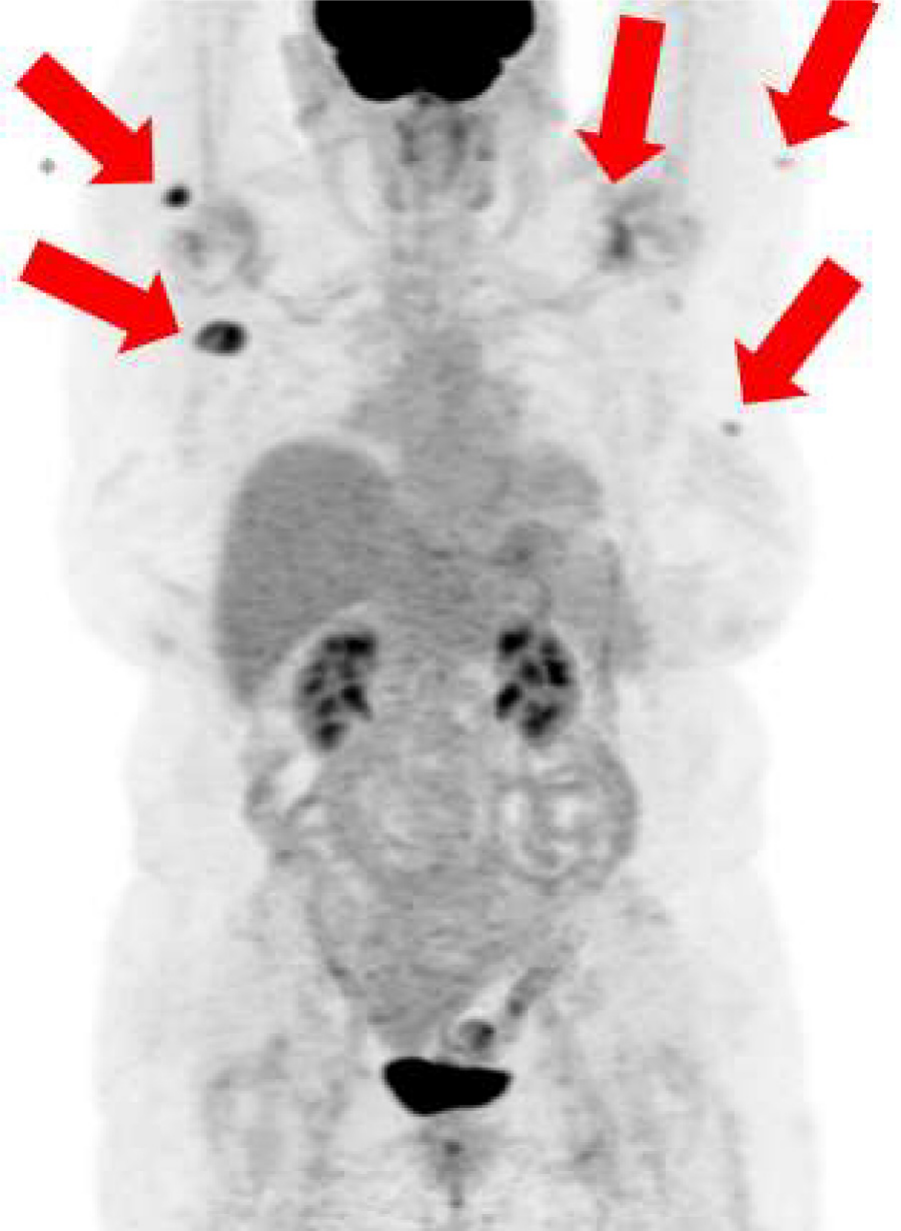
PET/CT demonstrated numerous suspicious lesions, including the left breast (SUV 1.9), left proximal arm laterally (SUV 2.1), left back laterally (SUV 2.8), proximal right arm/right shoulder posteriorly (SUV 7.2/1.1 cm), right back posteriorly– not imaged (SUV 1.8), and right back laterally posterior to the right scapula with clustered lesions (SUV 7.6).

The patient was subsequently referred to oncology. FDG PET-CT showed several cutaneous and subcutaneous hypermetabolic lesions (Deauville 4) suspicious for active lymphoma (Fig. 8). Dermatology performed a punch biopsy of a posterior shoulder plaque, confirming cutaneous B-cell lymphoma. Bone marrow biopsy showed a CD5-positive, kappa-restricted small B-cell population (approximately 6% of white cells), without a monotypic plasma cell component. The patient was considered to have an atypical presentation of Lugano stage 4 EMZL.

## Discussion

Breast lymphoma represents ∼0.5% of breast cancers. EMZL accounts for a minority of breast lymphomas (∼12%-20% in most modern series) and EMZL overall (∼2% present in the breast) [[4], [5], [6], [7]], which may explain radiologists’ bias toward benign etiologies when a superficial mass is visualized. The imaging appearances of EMZL and other breast lymphomas often overlap with benign entities and other malignancies, and can occur as a breast mass, enlargement of a lymph node, as well as changes to the skin or subcutaneous tissues [8,9]. These findings are often nonspecific, prompting the need for additional imaging maneuvers to better localize and characterize the abnormality.

When morphology is indeterminate on mammography, a BB plus tangential view that brings the skin into profile is decisive. [3]. In some cases, digital breast tomosynthesis and careful assessment of depth relative to the skin line add confidence, but this is not always specific. [10]. Ultrasound should document the lesion’s position relative to the epidermis, the presence of a dermal tract, margin characteristics, and Doppler flow. When a new or atypical superficial lesion lacks unequivocally benign features—or lacks a visible skin correlate—BI-RADS 4 and tissue sampling are appropriate, even when the target is subdermal.

The imaging differential for skin-based findings includes benign entities (epidermal inclusion/sebaceous cyst, Mondor disease) and malignant mimics (inflammatory breast cancer, angiosarcoma, dermatofibrosarcoma protuberans (DFSP), lymphoma). There are practical discriminators to differentiate between these diagnoses on imaging: cysts often have a dermal tract with a classic laminated keratin pattern [11,12]; Mondor disease have a noncompressible, beaded tubular structure without Doppler flow [13]; inflammatory breast cancer has dermal thickening and erythema [14]; angiosarcoma and DFSP are superficial masses with rapid growth and associated dermal discoloration [[15], [16], [17]]. The differential diagnosis is summarized in Table 1.

**Table 1:** Differential diagnosis of skin based findings encountered at breast imaging.

Diagnoses	Imaging findings by modality			
	Mammogram	Ultrasound	Magnetic resonance imaging	Contrast enhanced mammography
Lymphoma in the breast (primary or secondary).	Solitary or multiple, noncalcified oval/round mass(es) with indistinct or circumscribed margins.	Hypoechoic mass(es) with circumscribed or indistinct margins with posterior enhancement. Presence of internal vascularity.	T2 hyperintense homogeneously enhancing mass(es) with restricted diffusion and mixed kinetics.	Mass(es) with moderate to intense enhancement.
Invasive ductal carcinoma	Irregular mass with spiculated margins, with or without calcifications. The mass may be located within the subdermal space or within the fibroglandular tissue with dermal extension.	Hypoechoic irregular mass with spiculated margins with posterior acoustic shadowing. Presence of internal vascularity.	Irregular, heterogeneously or homogeneously enhancing mass with washout Type III kinetics.	Irregular or rim enhancing mass with heterogenous or homogenous enhancement.
Angiosarcoma (typically will have reddish, purplish, or bruise-like discoloration of the skin).	Primary angiosarcomas may present as a mass within the breast parenchyma with dermal extension. Whereas secondary angiosarcomas will typically have subtle asymmetric dermal thickening with or without an underlying parenchymal involvement.	Dermal thickening with nonspecific hypoechoic or heterogenous nonmass lesion within the dermal or subcutaneous tissue with or without an underlying hypoechoic mass.	Asymmetric dermal and subdermal thickening associated with T2 hyperintensity and enhancement demonstrating washout Type III kinetics. Primary angiosarcomas will typically have an underlying enhancing mass, also with washout Type III kinetics.	Enhancing asymmetric dermal thickening with or without an underlying enhancing mass.
Dermatofibrosarcoma protuberans	Oval or lobulated mass located close to the dermis without calcifications.	Oval or lobulated hypoechoic or heterogenous solid oval mass within the dermis or subcutaneous tissue with or without focal dermal thickening.	T1 iso- to hypointense, T2 hyperintense, oval or lobulated heterogeneously enhancing mass near the dermis.	Heterogeneously enhancing oval or lobulated mass close to the dermis.
Mondor’s disease (patients may feel a “rope-like” palpable mass).	Tubular “beaded” high density lesion located in the subdermal/subcutaneous region.	Tubular structure that is anechoic or isoechoic without internal vascularity. Typically, there are multiple areas of narrowing giving it a “beaded” appearance.	Superficial nonenhancing tubular structure corresponding to a thrombosed vein. There are variable internal T1 characteristics depending upon the thrombus age. Typically T2 hypointense with mild surrounding T2 edema.	Tubular nonenhancing density in the subdermal/subcutaneous region. There may or may not be a subtle linear enhancement surrounding the structure from inflammation.
Epidermal inclusion cyst	Circumscribed round or oval mass in the dermis which may or may not contain calcifications.	Anechoic or hypoechoic, round or oval cyst with posterior acoustic enhancement. Absence of internal vascularity.	T2 hyperintense, nonenhancing mass at the dermis. There may be rim enhancement if the cyst is inflamed.	Round superficial mass with minimal or no enhancement unless inflamed.

In our case, pathology showed CD-5 positive, low-grade B-cell lymphoma with plasmacytic differentiation. Although EMZL is usually CD5-negative, CD5 expression can occur and mandates correlation with fluorescence in situ hybridization studies (FISH) (no CCND1 or CCND2 rearrangement) and flow cytometry to exclude mantle cell lymphoma and small cell lymphoma (SLL) [18]. The largely cutaneous distribution, immunophenotype, and absence of LEF1 receptors, supported EMZL over these alternatives [19].

Although FDG PET/CT demonstrates variable avidity in EMZL, it is increasingly employed to distinguish systemic disease from primary cutaneous marginal zone lymphoma and is useful in identifying occult lesions. In our case, PET/CT revealed additional skin/subcutaneous targets with a Deauville score of 4. Additional histologic confirmation in marrow and skin support systemic disease and justify stage IV by Lugano criteria [20,21]. While the 2014 Lugano classification considers EMZL to be a non-FDG-avid disease, consistent with recently published standards, this patient’s imaging findings were FDG avid and the use of PET supported an understanding of several smaller lesions that were much less apparent on noncontrast CT consistent with disseminated disease [22].

## Conclusion

Superficial findings on screening mammography are common, yet are often being mistaken for benign skin lesions such as a skin mole. If these lesions have benign characteristic shapes such as round or oval lesions, it poses an even greater challenge to raise a mammographer’s suspicion/attention to these lesions. Radiologists must understand the diverse imaging findings of both benign and malignant subdermal lesions. Familiarity with an array of subdermal breast lesions can reduce false reassurance and streamline triage to appropriate systemic staging and care if necessary.

## Authors' contributions

Yu-Soon Aileen Park: Conception and design, Study analysis, Interpretation of data, Draft manuscript, Revise manuscript critically, Final approval, Agreement to be accountable for all aspects of the work. David Supeck: Conception and design, Study analysis, Interpretation of data, Draft manuscript, Revise manuscript critically, Final approval, Agreement to be accountable for all aspects of the work. Alan Zhu: Conception and design, Study analysis, Interpretation of data, Draft manuscript, Revise manuscript critically, Final approval, Agreement to be accountable for all aspects of the work. Laura Harper: Conception and design, Study analysis, Interpretation of data, Draft manuscript, Revise manuscript critically, Final approval, Agreement to be accountable for all aspects of the work. Richard Sharpe Jr: Conception and design, Study analysis, Interpretation of data, Draft manuscript, Revise manuscript critically, Final approval, Agreement to be accountable for all aspects of the work.

## Patient consent

Informed consent for publication of this case was obtained from the patient.
